# Diabetes

**Published:** 1905-06-03

**Authors:** 


### DIABETES.

**Treatment.**—As a large number of unreliable and unsuitable bread substitutes are in the market, Williamson[1] has drawn up the following four criteria by which a diabetic preparation may be judged:—1. It must be practically free from starch and sugar. 2. Its taste must be agreeable to the patient. 3. Its cost must be within the patient's means. 4. In the case of biscuits, they must not be too hard for the patient's teeth, which are often carious in diabetes. Any preparations should be tested for starch, before being ordered, with iodine 1, pot. iod. 1, water 100. This should give no blue colour, or only the very faintest. It should be also tested for sugar by the yeast test, often employed for testing urine. No preparation containing much starch or sugar should be used, as if the diet is not to be strict it is better to allow a little bread or potatoes. The following preparations are reliable:—1. Prepared from vegetable albumens: Roborat bread, aleuronat bread, gluten bread. 2. Prepared from nuts: Almond cakes, cocoa-nut cakes. 3. Prepared from milk albumen: Plasmon, protene, casoid. (1) Roborat contains fat in addition to proteid, but very little carbohydrate. It may be made into small rolls with butter, eggs, yeast, and a pinch of salt. Williamson has proved that sugar excretion diminishes markedly when Roborat is substituted for ordinary bread. It may be employed also mixed with protene or almonds. Its taste is very satisfactory. Aleuronat, mixed with cocoanut powder, may be made into cakes containing only a very small amount of carbohydrate. When freshly prepared most patients like them, but they will not keep more than a few days. They are best taken hot with butter. Sugar excretion is much diminished when this bread substitute is employed. Gluten bread is often very unreliable and also distasteful to many patients. All gluten preparations should be very carefully tested with iodine before they are permitted. (2) Almond flour and cocoanut powder both contain a little sugar, but this is destroyed by the yeast used to make them into cakes. They sometimes give rise to dyspepsia owing to the amount of fat they contain. This can be obviated by giving a little alcohol with the meals. (3) Plasmon is free from carbohydrate, but the biscuits are often too hard for diabetes. Protene is practically sugar and starch free. The bread and biscuits may be obtained ready made. The author has obtained satisfactory results from protene bread. Casoid bread and casoid meal-bread are both satisfactory preparations, and the biscuits made from the same substance are liked by many diabetics. The casoid preparations, unlike the others, cannot be prepared at home. The best plan is to allow the patient to choose for himself among the various reliable preparations, and to change from one to another when he wishes. In spite of Kaufmann's [2] opinion that no drugs are of any service in diabetes except opium and salicylic acid or their derivatives, and that of these opium is the only effective one in severe cases, many others have been favourably reported on from time to time. Tylecote [3] has recently collected the literature relative to uranium nitrate, which has been employed by Hughes, West, Duncan, and others. The author's conclusions are that the drug should be given in small doses, well diluted, and gradually increased. It causes an improvement in weight and subjective feeling of well-being, but no decrease in sugar excretion. Polyuria is occasionally decreased and pulse tension heightened. Falling weight, the appearance or increased amount of albumen in the urine, and increase of sugar excretion are indications for discontinuing the drug. Noel Paton [4] has experimented with adrenalin and thyroid extract on the sugar excretion in diabetics. Adrenalin chloride (1 in 1,000) was given subcutaneously to two patients suffering from a mild and severe form of diabetes respectively. The doses raised from ·5 cc. twice daily to ·75 cc. thrice daily. In both cases there was marked increase in sugar excretion and a less marked increase in proteid waste. A patient to whom ·5 gm. to 10 gms. of thyroid extract was given for six days developed symptoms of coma, without any modification of the sugar and nitrogen excretion. Allan,[5] commenting on two cases in which pancreas was transplanted without success in diabetes, considers that the attempt should only be made with the patient's consent when the case does not respond to drugs and dieting, when it is drifting into coma, and when acetonuria is absent. The sheep whose pancreas is used must be killed close to the place of operation, and its pancreas directly conveyed to the patient.

**The Nervous System and Diabetes.**—The pathological changes in the nervous system found in diabetes are slight but significant, according to Dickinson.[6] They consist of small hæmorrhages and extravasations in the central nervous system, enlargement of the perivascular spaces and distension of the central canal of the cord. They may be taken as indicating changes in the tone of the blood-vessels, and are no doubt secondary and not causal in character. The involvement of the nervous system is seen in several clinical facts. Engine-drivers, whose work makes great demands on the nervous system, are more liable to diabetes than the generality of men. Diabetes, or at any rate glycosuria, is certainly not infrequently observed in insane persons, especially in melancholics. Maudsley has shown that diabetes and insanity are found in different members of the same family; Dawson divides the cases into two groups—those in which mental disease arises in the course of glycosuria, which is rare, and those in which insane persons develop glycosuria, which is much more usual. Dickinson quotes also Raimon and Bond in support of this connection, and considers that Hale White has to a certain extent supported it while endeavouring to disprove it, as he admits that sugar was undoubtedly present in 4 per cent. of the patients he examined and possibly present in 10 per cent. Loss of the patellar reflex and areas of anæsthesia Dickinson considers as probably due to the irritant action on the nerves of the saccharin blood. Dickinson, while admitting that diabetes may be only a name for a group of diseases presenting one common symptom, inclines to the view that in many cases the cortex and medulla are primarily at fault and act through the liver, causing an excessive sugar production, not merely a failure in assimilation. He also notes the occurrence of diabetes after cerebral injuries and mental worries; with regard to injuries Tyson [7] says that blows on the back, legs, thorax, kidney, and liver may be followed by diabetes; Senator [8] points out that the damaged organ need not necessarily be one concerned in the production of glycosuria; while Schmitz[9] considers any nervous worry or mental shock may be regarded as the cause of a diabetes or increase that condition if it exists. Lorand [10] has recorded three fatal cases following fright and mental shock, and Von Noorden [11] gives a year as the period within which glysosuria must appear in order to establish its causal connection with shock. Kœrner [12] has collected 38 cases of operation in diabetics; in 13 the condition was slight and recovery ensued, in five it was moderate and the results were equally good, in 9 the disease was severe and of these 4 died—3 from coma and 1 from general weakness. Of 6 other unclassified cases all recovered. Kœrner considers that all cases for operation should be previously dieted and put on large doses of sodium bicarbonate, should be operated on early in the morning, and as rapidly as possible.

[1] Med. Chron., Jan. 1905, p. 228. [2] Birm. Med. Rev., Sept. 1904, p. 517. [3] Med. Chron., Sept. 1904, p. 379. [4] Scott. Med. Surg. Jour., Dec. 1904, p. 492. [5] Birm. Med. Rev., Sept. 1904, p. 517. [6] Lancet, Dec. 10, 1904, p. 1629. [7] Brit. Med. Jour., Oct. 8, 1904, p. 936. [8] Ibid. [9] Ibid. [10] Birm. Med. Rev., Sept. 1904, p. 511. [11] Brit. Med. Jour., loc. cit. [12] Birm. Med. Rev., Sept. 1904, p. 516.

*(To be continued.)*

